# The College of Nursing (Limited by Guarantee)

**Published:** 1920-12-25

**Authors:** 


### THE COLLEGE OF NURSING (Limited by Guarantee).

The quarterly meeting of the Irish Board was held at 54 Fitzwilliam Square on December 13, the Chairman, Dr. Peacocke presiding. Also present: Miss Adams (Dublin); Mrs. Clokey, Miss Curtin, Miss Gault, Miss Jack (Belfast); Miss Hill, Miss Michie (Dublin); Mrs. M'Whinney, Miss Musson (Belfast); Miss Thomas (Dublin); Dr. F. Dunne, Sir John Lumsden, and Sir John Moore. Apologies for non-attendance were received from Miss Bostock, Miss Chisholm, and Miss Rohde.

The resignation of Miss M. M. Robinson was accepted with regret.

Surgeon J. B. Moore, of Belfast, was co-opted a member of the Board to fill the vacancy caused by the death of the late Sir Alexander Dempsey.

The report of the Nurses' Club showed a gratifying rise in the number of nurses staying in the Club, the bedroom accommodation being frequently taxed to the utmost. In response to several requests, the Committee had finally agreed to extend the term of residence under certain circumstances, and in future to accommodate nurses wishing to reside in the Club for a period of three months or longer at reduced terms.

The Belfast delegates reported arrangements made for the holding of a public meeting in Belfast on January 12 with a view to the formation of a local Centre of the College for Belfast and district, the chair to be taken by Mr. R. J. Johnstone, F.R.C.S., and the meeting to be addressed by, amongst others, the Organising Secretary of the College, Miss Sheriff MacGregor.

The intervention by the Board on behalf of College members in Mill Street Union had resulted in satisfactory increases of salary. Other appeals on behalf of members were reported to be still in progress.

A scheme for attracting suitable candidates to the profession was outlined in the report of the Executive Committee, which was adopted.

It was agreed to open a fund for the development of the activities of the College in Ireland, such as the provision of scholarships, opportunities for post-graduate study, etc., subscriptions being invited from all who are interested in the higher development of the nursing profession.

Other important business having been transacted, the meeting adjourned.

### AT THE BRISTOL CENTRE.

Mr. Handley, President of the Labour Exchanges in Bristol, addressed a meeting of the local branch of the College of Nursing on the Unemployment Act. The meeting was held at the Bristol Royal Infirmary. Mr. Handley in a sympathetic speech made clear many of the intricate problems of modern industrial conditions, and all to clear the appalling instability of modern employment. The dominant note of his theme was calling on the employed to be willing to help the unfortunate by their ungrudging payment of the weekly contributions, that the country may be enabled to create a fund—a real insurance for the future which may lead to a more happy and prosperous England. The nurses greatly enjoyed the lecture, but some felt a doubt whether the official expenditure entailed might not prove too heavy to give the desired result.

Mr. Handley, on being questioned as to the method of administering unemployed grants to nurses, suggested that through their nursing organisations they should endeavour to establish their own professional exchanges.

At the close the question of "Commissioned Rank for Nurses" was discussed, and the matter being put to the meeting commissioned rank was, with the exception of one vote, approved unanimously.

The forthcoming election of the College Council was also considered, and the meeting closed with an interesting lecture on "Presence of Mind" by Dr. Annie Cornell.

### COLLEGE MEMBERS AND THE RECENT REFERENDUM.

Members are notified that acknowledgment of communications will be sent as soon as possible.

The extra work thrown on the office, and the difficulty, therefore, of posting immediate acknowledgments, it is sure will be understood.

